# Healthcare providers' approaches to sexual function in women with migration histories: a qualitative study using Andersen's behavioural model

**DOI:** 10.1186/s12939-026-02917-9

**Published:** 2026-06-18

**Authors:** Negin Mirzaei Damabi, Mumtaz Begum, Jodie C. Avery, Salima Meherali, Zohra S. Lassi

**Affiliations:** 1 Robinson Research Institute, College of Health, Adelaide University, Adelaide, Australia; 2 School of Public Health, College of Health, Adelaide University, Adelaide, Australia; 3 Adelaide Medical School, College of Health, Adelaide University, Adelaide, Australia; 4 Faculty of Nursing, University of Alberta, Edmonton, AB Canada

**Keywords:** Sexual function, Migrant, Refugee, Healthcare providers, Women

## Abstract

**Background:**

Sexual function (SF) remains a critically neglected dimension of Sexual and Reproductive Health (SRH), particularly for Women with Migration Histories (WMH), who face intersecting cultural, institutional, and systemic barriers to care. Despite global health frameworks positioning sexual wellbeing as integral to holistic health, SF is rarely integrated into routine SRH practice. This study examines healthcare providers’ approaches to SF in the clinical care of WMH in South Australia (SA).

**Methods:**

An exploratory qualitative design was employed, using semi-structured interviews with ten SRH providers in SA, including nurses, allied health professionals, and a gynaecologist. Participants were purposively sampled for their experience in caring for WMH. Data were analysed thematically using NVivo. An adapted version of the Andersen Behavioural Model of Health Services Use (ABMHSU) was applied to guide the development of the interview guide and structure the analysis across three domains: predisposing factors, enabling resources, and need-related factors.

**Results:**

Providers described limited engagement with SF in routine clinical care, which remained focused on reproductive control. They identified predisposing barriers operating within the clinical encounter, including taboos and stigma surrounding sexual health, marginalisation, mistrust, and communication difficulties shaped by cultural and linguistic distance. Enabling resources were undermined by Medicare ineligibility, financial hardship, interpreter-related concerns, and fragmented care pathways. Need-related barriers included system strain limiting timely responses and restricted access to culturally appropriate sexual therapy. Together, these interconnected barriers were perceived to reinforce the marginalisation of SF in SRH care for WMH.

**Conclusions:**

Incorporating SF into routine SRH care for WMH requires coordinated improvements at both the service and provider levels. At the service level, modifiable strategies include culturally tailored SRH literacy, interpreter-sensitive communication, system navigation support, and Medicare-funded, culturally adapted sexual therapy. At the provider level, addressing personal discomfort, insufficient training, and concerns about cultural appropriateness requires comprehensive, mandatory training in trauma-informed and culturally safe care, supported by centralised and accessible resources. Together, these interventions are critical to improving SRH service provision and utilisation, sexual autonomy, health literacy, consumer satisfaction, and overall quality of life for WMH.

**Supplementary Information:**

The online version contains supplementary material available at https://doi.org/10.1186/s12939-026-02917-9.

## Background

Despite decades of global advocacy for comprehensive Sexual and Reproductive Health (SRH), dimensions such as Sexual Function (SF) and pleasure continue to remain marginalised in policy and clinical practice [1, 2]. This neglect reflects enduring structural and normative challenges in recognising sexual wellbeing as an integral component of health [1, 2]. It is most evident in clinical discourse, which prioritises reproductive control and disease surveillance, while disregarding experiential dimensions such as desire, arousal, orgasm, and pain [3].

The World Health Organisation (WHO) defines sexual health as a state of physical, emotional, mental, and social well-being related to sexuality, encompassing not merely the absence of disease but the presence of pleasure, autonomy, and safety [3]. Within this positive sexuality framing, sexual function is understood broadly to include desire, arousal, orgasm, satisfaction, pleasure, and the absence of pain, situated within the wider experience of sexual wellbeing [4]. Yet, this holistic conception remains largely aspirational. Marginalisation is especially acute for women, who face disproportionately high rates of sexual difficulties and dissatisfaction and are frequently silenced or dismissed in clinical encounters [5, 6].

This systemic neglect is further underscored by population-level evidence. Epidemiological data reveal that 40 to 50% of women experience at least one sexual health concern in their lifetime [7–10], yet only approximately 20% seek professional assistance [10]. Despite the prevalence and complexity of these concerns, SF remains largely absent from routine clinical dialogue. In one United States survey of 3,807 patients, 54% expressed a desire to discuss sexual health with a provider, yet more than half of those who did were dissatisfied with the care received [11]. Similarly, findings from the REVIVE study (web-based, cross-sectional survey; *n* = 3,046), which focused postmenopausal women with self-reported vulvovaginal atrophy (VVA) symptoms, revealed that although 40% of women expected providers to initiate discussions about their symptoms, but only 19% reported that a provider had asked about their sexual health during a routine examination [12]. Even when women disclosed symptoms, many found that their concerns were minimised or met with discomfort [13].

This silence is not unidirectional. Healthcare providers often hesitate to initiate discussions about SF due to personal discomfort, insufficient training, and perceived cultural inappropriateness [14–16]. A survey of 222 physicians attending a sexual health education course found that fewer than half (48.3%) were experienced in managing sexual problems [17]. Another survey of urogynecologists (*n* = 471) revealed that although 51% considered screening for Female Sexual Dysfunction (FSD) to be very or extremely important, only 22% reported always screening, while nearly one-quarter admitted they rarely or never did so [18]. These findings underscore how provider-level discomfort and under preparedness reinforce the communicative barriers faced by patients.

For Women with Migration Histories (WMH), these challenges are significantly compounded [6, 19]. Cultural stigma, migration-related trauma, language barriers, and healthcare inaccessibility intersect to create profound structural and interpersonal constraints on care [19]. Although these women may experience heightened vulnerability to sexual dysfunction due to both psychosocial stressors and systemic exclusion, their concerns are seldom addressed in either clinical encounters or academic research [19]. The dominant discourse in SRH remains focused on family planning, infectious disease, or violence prevention, thereby rendering concerns about sexual satisfaction, desire, or intimacy as peripheral or illegitimate, positioning concerns related to sexual satisfaction, desire, and intimacy as peripheral or clinically irrelevant [2].

Despite a growing emphasis on culturally competent sexual health communication, there remains a marked absence of research specifically examining how healthcare providers engage with SF concerns in this population. This study addresses this gap by examining healthcare providers’ approaches to SF in the clinical care of WMH in South Australia (SA). In doing so, it aims to foreground sexual wellbeing/function as a legitimate health concern and interrogate the systemic, cultural, and professional barriers that perpetuate its neglect.

## Methods

### Study design

A qualitative study employing an exploratory, descriptive design was conducted using semi-structured interviews [20]. The design was selected to allow in-depth exploration of how providers approach SF in routine SRH care for WMH and to surface the cultural, institutional, and practice-level factors shaping their engagement with this aspect of care.

### Participant recruitment

Participants were individuals involved in delivering SRH services in SA, recruited through an intentionally broad strategy to capture diversity in terms of professional roles and demographic characteristics (gender, age, country of birth, and ethnicity). This included gynaecologists, General Practitioners (GPs), sexual health specialists, nurses, midwives, allied health professionals (e.g., psychologists, social workers, physiotherapists), and community service providers such as counsellors and caseworkers. Recruitment sought breadth across professional roles and demographic backgrounds, although the final sample composition was shaped by who responded.

Convenience sampling, supplemented by snowball sampling, was used [21]. Convenience sampling drew on networks at the Robinson Research Institute, Adelaide University, and Healthy Development Adelaide, where SRH expertise was concentrated. Snowball sampling was used in later stages of recruitment, when initial participants referred colleagues with relevant experience. Recruitment was conducted through targeted social media outreach via these institutional networks and through a dedicated project website, RISE (Refugee and Immigrant Sexual Health Endeavour), which centralised study information, eligibility, consent documentation, and interview scheduling. Recruitment occurred between January and May 2025.

The target sample size of 10–15 providers was informed by qualitative literature on information richness in SRH research [22] Data sufficiency was assessed iteratively through team discussions during data collection, with recruitment concluding when new interviews were no longer generating substantive new codes and the three analytic domains were richly represented in the data [23].

Despite extensive outreach, engagement of senior medical specialists, particularly gynaecologists and GPs, was limited. This limitation, shaped by the convenience-based design, affects the transferability of findings and is revisited in the Limitations section.

### Conceptual framework

This study was guided by Andersen’s Behavioural Model of Health Services Use (ABMHSU), a widely recognised theoretical framework in health services research for understanding the factors that shape healthcare access and utilisation [24, 25]. Originally developed in the context of the U.S. National Health Survey, the model has undergone multiple refinements to accommodate evolving health policy contexts and service delivery realities [26]. It remains grounded in three primary domains: predisposing characteristics, enabling resources, and need-related factors. These domains are designed to capture a comprehensive array of influences that shape whether, how, and why individuals engage with healthcare services [27].

Predisposing characteristics include demographic, attitudinal, and socio-cultural factors that influence individuals’ likelihood of seeking care, such as beliefs about health, stigma, or prior experiences. Enabling resources refer to the logistical and structural conditions that affect healthcare access, including socioeconomic conditions (e.g., financial resources, housing stability), availability of services, interpreter support, healthcare entitlements, and provider training. Need-related factors encompass both perceived health concerns and clinically identified conditions that drive care-seeking and determine service engagement [24].

This framework was selected for its strong explanatory power and adaptability to diverse populations, particularly in studies examining health inequities among Culturally and Linguistically Diverse Groups (CALD) [28]. Although ABMHSU is conventionally applied to explain individuals’ health service use, in this study it is used to interpret provider accounts of the barriers shaping WMH’s access to and utilisation of SRH care. Providers function as informants whose clinical experience offers situated insight into the predisposing, enabling, and need-related factors influencing WMH’s engagement with services. The framework therefore organises provider observations of patient-level access barriers, rather than treating providers themselves as the subjects of the model.

### Data collection

Semi-structured interviews were conducted in English and either in person or via secure online platforms, based on participant availability and preference. The data collection period spanned between January and May 2025, with interviews lasting approximately 45 to 60 min. All interviews were audio-recorded with participants’ informed consent to ensure accurate data capture and transcription integrity.

At the start of each interview, sexual function was operationally defined in line with the positive sexuality framing of the WHO definition of sexual health to establish shared understanding between interviewer and participant [3]. SF was defined as encompassing desire, arousal, orgasm, satisfaction, pleasure, and the absence of pain, and as situated within the broader experience of sexual wellbeing rather than reduced to physiological response [4]. This shared working definition framed all subsequent discussion in the interviews.

The interview guide comprised open-ended questions informed by ABMHSU, designed to elicit comprehensive insights into healthcare providers’ experiences in addressing SF concerns among the this population. The complete set of interview questions is provided in Supplementary File 1. Participants were reimbursed for their time in accordance with the guidelines issued by the National Health and Medical Research Council (NHMRC) on participant payments in research [29].

### Data analysis

Transcription, coding and thematic analysis of the interview data were performed following the six-phase framework outlined by Braun and Clarke [30]. This approach provided a structured yet flexible method for identifying, coding, analysing, and reporting patterns within the data, aligning with the study’s exploratory design.

Following transcription, the research team engaged in data familiarisation through multiple readings of the transcripts, enabling immersion in the content and the identification of preliminary patterns. Data coding was facilitated using NVivo software (version 15), which enabled systematic organisation and segmentation of the data into meaningful units [31].

The coding process proceeded in two stages. Initially, codes were developed inductively from the data to capture healthcare providers’ experiences, perceived challenges, and recommendations for delivering sexual health care to WMH.

ABMHSU then guided the thematic organisation of the data, providing a theoretical lens through which emergent patterns were categorised and interpreted. This framework supported the analytic process by offering a coherent structure for mapping themes across predisposing characteristics, enabling resources, and need-related factors [24].

Themes were iteratively discussed among the research team to maintain consistency and ensure that findings reflected participants’ narratives accurately. Regular team meetings were held to review coding decisions, resolve discrepancies, and refine thematic interpretations. Exemplar quotations were selected to illustrate each domain while maintaining participant confidentiality through the use of pseudonyms and removal of identifying details.

### Ethical considerations

Ethical approval for this study was obtained from the Human Research Ethics Committee at the University of Adelaide (Approval: H-2024-011) on 30 January 2024. Informed consent was obtained from all participants prior to data collection, ensuring they were fully informed about the study’s objectives, procedures, and potential risks. Participants were explicitly informed that their involvement was voluntary and that they could withdraw from the study at any stage without consequence.

Confidentiality and anonymity were rigorously maintained throughout the research process. Participant identities were protected by assigning unique identification codes, and all identifying information was removed from transcripts to safeguard privacy. Data were securely stored on password-protected systems of the university accessible exclusively to the approved research team, adhering to institutional data confidentiality and data management protocols.

To acknowledge the time and effort involved, participants received a $100 gift card after the interview. This approach aligns with NHMRC guidelines, which support modest reimbursement for time, effort, and inconvenience, provided it does not constitute coercion or undue influence [29].

### Reflexivity and researcher positioning

The research team comprised individuals with migrant backgrounds, which significantly facilitated culturally sensitive and empathetic interactions during data collection. This shared background enabled the researchers to establish rapport and foster open discussions around the sensitive topic of sexual health, creating a trusting environment where participants felt comfortable sharing their experiences. The team’s cultural insight proved invaluable in navigating complex and potentially stigmatised conversations, thereby enriching the depth and quality of the data collected.

To maintain analytical rigour and mitigate potential biases, the team engaged in continuous reflexive practices. Researchers actively reflected on how their personal experiences and cultural backgrounds might shape data interpretation, ensuring that the findings remained firmly grounded in participants’ narratives rather than researchers’ preconceptions and personalities. This commitment to reflexivity underscored the team’s dedication to maintaining analytical integrity and accurately representing participants’ voices.

Despite the team’s extensive efforts to engage participants across diverse professional areas, including gynaecologists, sexual health specialists, and GPs, recruitment presented significant challenges. The team leveraged every available network, including institutional affiliations and professional connections, demonstrating exceptional persistence and adaptability in outreach strategies. The creation of the RISE website further exemplified the team’s innovative approach to participant engagement, offering a centralised, multilingual platform for study information and recruitment.

Nevertheless, convincing healthcare providers to participate proved difficult, highlighting a broader challenge within the research area. Future studies should consider implementing targeted incentives or strategic partnerships to facilitate greater engagement from healthcare professionals. Addressing these recruitment barriers is critical for capturing a broader spectrum of provider perspectives and ensuring comprehensive data collection in research focused on culturally diverse populations.

## Results

The study involved 10 healthcare providers, comprising one gynaecologist, four nurses, and five allied health and community service professionals (including a psychologist, clinical psychologist, social worker, and community counsellor), all of whom were actively engaged in delivering SRH to WMH in SA. The sample comprised one man and nine women from diverse backgrounds: two born in Australia, one in the UK, two in East Asia, three in South Asia, and two in the Middle East.

Thematic analysis identified a set of interrelated barriers, reported by providers, that shape WMH’s access to and utilisation of SF-related care within SRH services in SA. Findings are organised according to the three core domains of ABMHSU and draw on providers’ situated observations of the cultural, institutional, and clinical dynamics influencing WMH’s engagement with care. An overview of these themes and subthemes is presented in Table 1.

**Table 1:** Summary of themes and subthemes mapped onto the andersen behavioural model of health services use (ABMHSU)

Abmhsu domains	Themes	Subthemes
Predisposing characteristics	Cultural and perceptual barriers to WMH’s SRH Engagement	• Cultural taboos and silences in SRH communication • Intersectional exclusion of marginalised identities • Fear of community surveillance and confidentiality breaches • Health literacy and cultural misalignment in SRH
Enabling resources	Structural and institutional constraints	• Legal, financial, and policy gatekeeping • Fragmented and incoherent service delivery • Limited provider training and cultural competence
Need factors	Complex care needs and therapeutic gaps	• Health system strain is limiting timely SRH responses to WMH’s SRH needs • Unmet therapeutic needs in complex SRH cases

### Predisposing characteristics: cultural and perceptual barriers to WMH’s SRH engagement

Healthcare providers, drawing on their interactions with WMH, emphasised that cultural norms surrounding sexual health, gender roles, and reproductive practices frequently discourage women from seeking assistance, disclosing sensitive information, or asserting sexual agency. These pressures often manifest as shame, fear of judgement, and internalised stigma, particularly regarding sexual violence, contraception, and autonomy.

At a systemic level, providers observed that a standardised, one-size-fits-all approach to service delivery fails to address the complex, intersecting needs of women facing trauma, domestic violence, or mental health concerns. This misalignment leads to missed opportunities for intervention and support:

My biggest concern is the one-size-fits-all approach. We have a system that provides the same services for everybody, not realising that people are not the same. That’s how we create inequalities when one system has one way of working, providing services across different populations without readjusting or readapting to the specific needs of women. (Nurse, Middle Eastern)

### Cultural taboos and silences in SRH communication

Providers reflected on how the topic of sexual health is treated as taboo within clinical encounters with WMH, making it difficult for conversations about SRH needs to be initiated. One provider described how cultural norms inhibit open dialogue about intimate matters in clinical settings, observing that sexual agency was rarely a frame of reference invoked in these encounters. Providers noted that, in their experience, expectations of privacy around sexuality and consent were sustained by both women and clinicians, contributing to reluctance on both sides to open the conversation:

The cultural values and beliefs are the biggest problem in women seeking help. Specifically, around a taboo topic such as sexual health. They come from a culture where they only seek help when it crosses. They don’t really seek help for nothing. Talking about sensitive issues, personal issues, issues that they’ve trained to keep only between them and their partner or family. They don’t share this with strange people. (Nurse, Middle Eastern)

For survivors of sexual harassment or assault, the stigma associated with sexual violence is particularly pronounced, leading to silence:

I know so many women who were victims of sexual harassment, and they didn’t say anything. It’s hard enough for English-speaking women, let alone for women who don’t speak English. They internalise the problem and the stigma associated with it. (Nurse, Middle Eastern)

Cultural stigma is not confined to patients. Providers acknowledged their own discomfort and internalised stigma as significant barriers to meaningful engagement. Overcoming these personal limitations was deemed essential for effective communication:

I always believe that they [health care providers] have to overcome their own shame and stigma before they can talk to somebody else. If I’m sitting here and I’m talking about sexual reproductive health while I am feeling uncomfortable, you are going to sense it. You’re going to pick it up. If I am not comfortable with what I’m talking about, nobody else is going to be comfortable about it. (Clinical psychologist, South Asian)

Providers also described how cultural norms around contraception, condom use, and sexual relationships shaped what could and could not be discussed in clinical encounters. They reported observing perceptions among some patients, including stigma associated with menstrual suppression and the framing of condom use as signalling mistrust or undermining masculinity, which providers felt constrained women’s reproductive autonomy and the openness of clinical dialogue:

Sometimes people have a partner here and another back home, and the other partner doesn’t know about it, or sometimes it is acceptable in their culture. We have to be sensitive to these cultural dynamics while also explaining that having multiple sexual partners can increase STI risks. (Community Counsellor, South Asian)

### Intersectional exclusion of marginalised identities

While stigma and discomfort often suppress discussions about SRH, internal biases and misconceptions further marginalise WMH whose identities diverge from dominant cultural norms. Providers acknowledged that addressing these biases is essential for creating an inclusive clinical environment:

You can’t really have a conversation with a sex worker if you don’t know what they go through. People think that all sex workers are doing it because they’re poor or desperate, but many do it by choice and take a lot of pride in their work. This is your internal stigma that you have to unpack. (Clinical psychologist, South Asian)

For LGBTQI + WMH, the intersection of cultural conservatism and sexual identity creates formidable barriers to accessing SRH services. Providers observed that cultural attitudes towards non-heteronormative identities exacerbate these challenges:

LGBTQI+, migrant, and refugee women may encounter additional barriers due to cultural attitudes towards non-heteronormative sexual identities. In many cultures, conservative views prevail, making it even harder for these women to seek support or discuss sexual health issues. (Community Counsellor, South Asia)

Certain cultural practices also complicate SRH care, particularly when women hesitate to disclose experiences that may be perceived as culturally sensitive or stigmatising. Female genital mutilation (FGM) was identified as a significant but underreported issue, with potential medical risks arising when healthcare providers are unaware of a patient’s history:

I’ve met quite a few women who have experienced genital mutilation, but nobody has ever disclosed it to me prior to a surgical procedure. It’s only when we go to the theatre and can’t proceed that we find out. And by then, they’ve already started on medication [Misoprostol], and it gets very tricky. (Nurse, Australian)

### Fear of community surveillance and confidentiality breaches

For many WMH, trust is a critical element in accessing SRH services. Establishing a sense of safety and assurance that personal information will be protected is essential for meaningful engagement. However, concerns about breaches of confidentiality often create significant barriers, particularly within small or tightly-knit communities. Providers described trust-building as a foundational step, without it, WMH are unlikely to disclose sensitive concerns or pursue necessary care:

Trust matters… You need to have heart and compassion… It means leaving yourself on the side and really being that person, trying to understand them… That skill doesn’t come just because somebody attended the training… We need to understand the balance between being task-oriented and relationship-oriented. (Nurse, Middle Eastern)

Despite confidentiality protocols, many women remain fearful that their information could be disclosed to family, community members, or healthcare staff from their own cultural background. This fear is especially acute among those reliant on interpreters, who, while intended to bridge language gaps, can intensify concerns about privacy, particularly when drawn from the same cultural or ethnic group:

It’s very subjective. We’ve had cases where people want to see a doctor who speaks their language because of the cultural context and because some words can’t be properly translated… But then there’s the issue of the interpreter. You don’t know who that third person is. They don’t want to go to someone from their community because of the hierarchy and fear of being recognised… If it’s a general health issue, they might trust someone from their community. But when it’s sexual health, they’re hesitant to talk to someone they know. (Clinical Psychologist, South Asian)

For some, the fear of recognition by a healthcare provider or interpreter is so intense that they avoid care altogether or seek services in distant locations. This perceived threat of exposure not only undermines care quality but also perpetuates silence and unmet health needs.

Providers also highlighted the interpreter’s dual role in clinical encounters; not just as a resource for patients, but as a vital conduit for clinicians to accurately understand patients’ experiences and cultural contexts:

I always ask healthcare providers who is the interpreter there for, you or the client? Most say, ‘For the client.’ But no, the interpreter is for me. They’re the bridge that helps me understand the client’s context, experiences, and needs. Without that, I’m just guessing.

This dual function becomes particularly complex in SRH settings, where communication must be precise and culturally sensitive. Providers noted that when interpreters lack training in SRH terminology or cultural nuance, miscommunication risks increase, further eroding trust.

### Health literacy and cultural misalignment in SRH

While trust and confidentiality are essential to fostering open dialogue in SRH care, limited health literacy and cultural misalignment present additional barriers that significantly influence WMH’s experiences. Providers emphasised that when cultural norms diverge from mainstream health frameworks, the consequences can include misdiagnosis, missed interventions, and ineffective care.

One example involved domestic violence cases going unrecognised because the language used by women to describe their experiences did not match clinical definitions of abuse. Providers noted that some women described sexual coercion or financial control without explicitly naming it as abuse, leading to missed opportunities for support:

She [one healthcare consumer] was a victim of war, diagnosed with mental illness, pregnant, and living in a domestic violence situation. But she didn’t name it as abuse. She said, ‘My husband is not abusive,’ but he was demanding sex without her approval, and she thought it was her role. The service assessed her and concluded there was no domestic violence because she didn’t say it outright. (Nurse, Middle Eastern).

This disconnect illustrates a critical gap in cultural competency within service delivery. When systems rely on standardised definitions, they risk overlooking culturally specific expressions of violence, excluding those whose experiences do not align with dominant frameworks.

Providers also reflected on how the SRH literacy of some of the women they cared for had been shaped by limited access to comprehensive sex education in conservative or conflict-affected settings of origin. They reported encountering women for whom sex had been framed solely in reproductive terms, with limited prior exposure to discussions of sexual agency, contraception, or reproductive rights:

I think for many women from diverse cultural backgrounds, sex is often something that its only purpose is to procreate. I’ve met women who legitimately had absolutely no idea that sex could make a baby, and they’re very confused that they’re now in this position where they are trying to make a choice as to whether they want to parent or not. (Nurse, Australian).

Providers expressed concern that, in their clinical experience, gaps in foundational SRH information among some women they cared for had downstream consequences for unintended pregnancies and for women’s ability to recognise coercive behaviours or assert reproductive autonomy in their relationships:

We have had some beautiful women come through, but very ignorant. They’ve just not received any education on their bodies, how to get pregnant, or even how they’ve become pregnant. Some have said, ‘I’ve never had sex with a man,’ even though they’re pregnant. It leaves them incredibly vulnerable. (Nurse, Australian)

Such health literacy gaps place significant strain on frontline providers, who must simultaneously deliver clinical care and foundational education. In the absence of culturally adapted literacy interventions, WMH may remain unaware of essential SRH information, perpetuating cycles of misinformation, vulnerability, and unplanned pregnancy.

### Enabling resources: structural and institutional constraints

This theme examines the systemic and institutional conditions that facilitate or hinder WMH’s access to SRH services. Healthcare providers identified three critical dimensions of enabling resources influencing service provision: financial and legal accessibility, system integration, and service coordination.

### Legal, financial, and policy gatekeeping

Healthcare providers described a complex set of structural barriers restricting access to SRH services for WMH in SA. These barriers, embedded within the broader healthcare system, act as gatekeeping mechanisms, limiting service utilisation based on legal status, financial insecurity, and policy exclusions. Although these constraints are systemically entrenched, their impact varies across different migrant cohorts, including international students, skilled migrants, and refugees or asylum seekers, each facing distinct legal, economic, and social challenges.

The most immediate of these constraints arises from financial hardship, which disproportionately affects WMH without Medicare eligibility or access to subsidised medicines under the Pharmaceutical Benefits Scheme (PBS). Participants consistently emphasised that the cost of SRH services, including routine screenings, vaccinations, and follow-up care, presents a significant barrier, especially for those lacking stable income or familial financial support. Women on temporary visas were identified as particularly vulnerable to financial exclusion:

Financial can be quite a problem for them. Sometimes they’re not working, or they may be stay-at-home mums and don’t have access to finances, or it’s only what they’re given. (Nurse, East Asian)

For these women, the lack of subsidised services compounded existing financial strain, forcing them to pay out-of-pocket for essential care typically covered by public funding. Providers expressed particular concern regarding the absence of subsidies for preventive services such as screening for sexually transmitted infections (STIs) and human papillomavirus (HPV) vaccination, both of which are vital for early detection and prevention of long-term sexual and reproductive health complications:

Not subsidised screening for STI and vaccination, particularly from a sexual health perspective. So, HPV, and hepatitis B. This is a significant gap. (Sexual Health Specialist, South Asian)

Financial barriers were especially pronounced for women without access to public healthcare, prompting some to seek more affordable treatment overseas. This cross-border health-seeking extended beyond SRH services, driven by the perceived affordability of care in less regulated health systems. Providers observed that women often travelled to their countries of origin for basic medical and dental care, exposing themselves to potential health risks due to inconsistent medical standards and inadequate infection control:

Everything is very expensive in Australia. People just visit their hometowns, and they think they can just get everything done over there. People don’t understand it could put them at risk. (Nurse, East Asian)

As financial barriers limited access to care, legal and policy exclusions created additional constraints. For WMH without permanent residency or Australian citizenship, visa-related fears emerged as a major deterrent to seeking SRH services. Providers noted that women with insecure immigration status often refrained from disclosing sensitive health information, including diagnoses such as HIV, due to concerns about potential repercussions for their visa applications:

Some women are afraid to seek help due to concerns about their visa or migration status. They think that if the government finds out that I have HIV, for example, it might affect my visa status. (Community Counsellor, South Asian)

For refugees and asylum seekers, the intersection of financial hardship and legal precarity created a particularly complex landscape of exclusion. Providers reported that these women often faced overlapping challenges, including trauma, unstable income, and limited legal protections, all of which impaired their ability to access and navigate SRH services:

When we talk about migrant populations, there are sort of three big cohorts that we talk about. There are international students whose health is managed through insurance, but they often worry about whether their parents will find out if they get STI testing. Then we have skilled migrants who may have Medicare and financial stability but still struggle to navigate the system. Lastly, we have refugees and asylum seekers whose experiences are completely different; they arrive traumatised, often without choice, and with complex healthcare needs. (Clinical Psychologist, South Asian)

Such structural disparities meant that even when services were technically available, they were not equitably accessible. For women already navigating the burdens of trauma, displacement, and precarious legal status, SRH needs were frequently deprioritised or deferred. In this way, structural gatekeeping, through legal, financial, and policy constraints, functioned not only to restrict access but also to reproduce exclusionary patterns that deepened existing health inequities.

### Fragmented and incoherent service delivery

Healthcare providers emphasised that fragmented service delivery, inconsistent referral pathways, and a lack of integrated care significantly obstruct WMH’s access to SRH services. Participants explained that navigating this disjointed system often resulted in missed appointments, reduced continuity of care, and heightened distress for patients already facing cultural and linguistic challenges.

Continuity of care was particularly problematic for patients with chronic health conditions such as HIV, where consistent monitoring is essential. One provider described how a patient disengaged from HIV treatment due to being seen by multiple doctors under a rotating roster system:

One of my clients used to have three-monthly and sometimes six-monthly appointments. She stopped going because previously, it was only one doctor. Now, they have a roster system, and she sees whoever is available on the day. She doesn’t feel comfortable talking to different doctors every time, different attitudes, different approaches. She doesn’t trust that they even have her history in front of them. And that’s very true. Sometimes, they don’t have time to go through it. (Community Counsellor, South Asian)

This distress was compounded by language and cultural barriers, further eroding trust in the system. The provider had to refer the patient to another hospital to secure more consistent care, an option not readily available to all.

Systemic fragmentation also affected broader service coordination, particularly regarding interpretation and referral. Providers reported that when interpretation services were unavailable, some clinics chose to send patients away rather than refer them to more accessible alternatives, thereby intensifying disruption in care continuity:

If somebody comes to them [healthcare provider] and they don’t have interpretation services available, they will just tell them to come next time instead of referring them to another organisation that has this ability. There should be more coordinated referral pathways between sexual health services and other services, like domestic violence services and refugee support agencies. (Health Program Manager, South Asian)

In response to these challenges, providers advocated for integrated care models that co-locate SRH services such as STI testing, contraception, and antenatal care into single, coordinated sites. Participants highlighted that such models would be particularly advantageous for WMH, who often struggle to navigate multiple disconnected service points across varied locations.

### Limited provider training and cultural competence

These structural challenges in service delivery were compounded by workforce-related barriers. Providers described how limited training and inconsistent access to culturally appropriate resources further undermined their ability to deliver effective, equitable SRH care to WMH. This lack of system-wide support deepened the fragmentation of services and eroded trust in the healthcare system. Healthcare providers consistently reported discrepancies between the availability and accessibility of training, observing that despite increasingly diverse patient populations, SRH-related training specific to WMH remains optional, inconsistent, and reactive. As a result, many providers felt underprepared to address the complex SRH needs of these groups.

Participants stressed the need to integrate trauma-informed and culturally safe care into core professional development frameworks, rather than relegating such training to occasional or situational sessions:

We need to be prepared to suit the needs of everyone coming through our doors, not just the mainstream Australian population. Lack of training in trauma-informed and culturally safe care service provision, particularly when it comes to sexual health, consent, and sexual health reproduction. (Health Program Manager, South Asian)

However, training often occurred only in response to specific incidents rather than being embedded in routine capacity-building. One provider illustrated this reactive model, describing how a session on kinks and fetishes was organised only after a staff member expressed discomfort:

We had a training yesterday. One of the staff members was not very comfortable talking about kinks and fetishes, so we organised a sexologist to come in and train the whole team. But that kind of training isn’t routine. (Clinical Psychologist, South Asian)

In addition to a lack of structured training, managers and supervisors cited difficulties in accessing centralised, consistent resources to support professional learning. Even where materials exist, they are often dispersed across platforms, limiting usability and integration into practice:

Resources are available, but they’re scattered across different platforms. Sometimes it’s quite hard for professionals to find what they need. You really have to make an effort to track them down. (Community Counsellor, South Asian)

This fragmentation not only impeded training efforts but also left providers feeling unsupported in maintaining cultural competence. While organisations such as Shine SA and SA Health do offer SRH-related materials, including translated resources, providers found these tools insufficiently consolidated and difficult to access:

In the South Australian context, Shine SA offers various resources on sexual and reproductive health. And I know there are some government agencies like SA Health that provide translated resources on specific topics like sexual health. But I would say they are quite scattered across different platforms. Sometimes it’s a bit hard for professionals to find what they need. You really have to make an effort to track them down. (Social worker, East Asian)

The absence of centralised, easily accessible training resources compounds the existing service delivery gap. Healthcare providers are left to navigate a fragmented educational landscape independently, which strains their capacity to remain informed about best practices and hinders the delivery of culturally competent SRH care to WMH.

### Need factors: unmet SRH needs and service gaps

This theme examines providers’ observations of unmet SRH need among WMH, where health system capacity and the availability of culturally appropriate therapeutic support fall short of the complexity of women’s needs. Providers described two interrelated barriers shaping how WMH’s needs are recognised and responded to: system strain limiting timely care, and the absence of culturally appropriate therapeutic services.

### Health system strain is limiting timely SRH responses to WMH’s SRH needs

Providers described how mounting pressure on GPs limits WMH’s timely access to SRH care. GPs are expected to manage an increasingly wide spectrum of concerns, including SRH assessments, trauma screening, mental health, and chronic disease management, often within constrained consultation times and without adequate remuneration. Providers reported that this systemic strain produced missed opportunities for comprehensive SRH care, particularly for WMH whose needs require longer consultations and trust-building. The prevailing expectation that GPs function as the primary contact for holistic SRH provision was critiqued as a systemic flaw. Providers expressed concern that the volume of responsibilities assigned to GPs significantly undermines their ability to respond effectively to the complex SRH needs of WMH:

There is enough training, there are enough resources. If people like to take them on board. But GPs, they are very, very busy. Everyone wants them to do sexual health assessments, sexual abuse assessments, mental health assessments and …. There’s so much that is put on GPs that they’re also like, ‘What else do you want us to do?’ (Clinical psychologist, South Asian).

This systemic overload has led to disengagement among some GPs, despite policy-level emphasis on their role in SRH care. Providers highlighted a disjunction between institutional expectations and the practical realities of general practice, including insufficient time allocations and lack of financial incentives:

I have given up on the issue of GPs because the literature keeps talking about GPs need to do this, GPs need to do that. But the system doesn’t support GPs to do it. No monetary remuneration, no time. There are a lot of issues, and sometimes they’re not the best. (Nurse, Middle Eastern).

This gap between policy intent and operational support not only limits the capacity of GPs to deliver consistent SRH care but also diminishes the potential for early intervention, particularly for WMH whose needs are often compounded by trauma, cultural stigma, and complex socio-environmental contexts.

### Unmet therapeutic needs in complex SRH cases

While education is a key component of SRH service provision, healthcare providers emphasised that it is insufficient for WMH whose SRH concerns are shaped by trauma, coercion, or cultural expectations. In these cases, sexual therapy and therapeutic group interventions were identified as essential to comprehensive care. However, such services remain largely inaccessible due to current funding constraints:

We need more than just education. It needs therapy [sexual therapy] because many migrant refugees come from trauma, war, and cultural issues that affect their sexual function. But where is that support under Medicare? (Nurse, Middle Eastern)

The absence of Medicare-funded sexual therapy services leaves critical care gaps, particularly for women whose experiences of trauma and SF difficulties require culturally sensitive, context-specific therapeutic responses. Without targeted funding, providers are left to manage complex needs with limited resources, compromising the quality of care.

Participants highlighted the potential of group therapy models to foster peer support, trust, and culturally grounded dialogue, creating safe environments for disclosure and healing. One nurse described how therapeutic sessions enabled women from a war-affected, marginalised community to articulate experiences that had long remained unspoken:

I used to run a therapeutic group for [a war-affected, marginalised] group. For the first few sessions, we only talked about what care means and how we know when someone is genuinely supportive. One woman said, ‘I hate sex. I give my husband a million excuses to go to bed early.’ It was the first time she had ever said it aloud. It took many sessions before she could even consider seeking help. (Nurse, Middle Eastern)

Despite evident need, funding limitations severely restrict the implementation of these services. Providers frequently reported delivering care beyond their official roles and resourcing, straining their capacity to meet demand:

A lot of times people are doing more than what the government is paying them to do, GPs, nurses, NGOs, they’re all going above and beyond their resources. If you ask me, are we doing enough? Yes, more than enough. Are we doing enough within our resources? Absolutely not. (Clinical psychologist, South Asia)

The absence of targeted financial support particularly affects WMH, whose SRH needs are often entangled with trauma, gendered violence, or coercion. Providers called for expanded Medicare eligibility and policy reform to enable access to sexual therapy, group interventions, and culturally tailored SRH services:

When it comes to government, there should be improved policy and funding structures. There should be targeted funding, and Medicare eligibility needs to be expanded to cover sexual therapy and therapeutic groups. (Health program manager, South Asian)

## Disscussion

SF remains one of the most under-researched and conceptually marginalised areas of women’s health, particularly for WMH who face intersecting layers of systemic exclusion [1, 32]. Although frameworks such as those developed by WHO promote a holistic conception of SRH that encompasses pleasure, agency, and satisfaction, healthcare systems continue to prioritise reproductive control and disease prevention, thereby omitting SF from routine care [2].

Providers in this study described a persistent disjunction between international policy rhetoric and clinical realities, in which SF remained absent from both professional training and the operational structures of SRH service delivery, particularly in services accessed by WMH.

This gap was consistently reflected in providers’ accounts of their own practice. Even in services where SF was nominally within scope, providers described discussions of desire, arousal, and satisfaction as rarely prioritised, often displaced by medically sanctioned concerns such as contraception or infection control. This marginalisation signals a broader institutional discomfort with framing sexuality as a component of wellbeing rather than a site of risk. It also reflects the entrenchment of a biomedical paradigm that privileges measurable, pathologised aspects of health over subjective, relational, or experiential concerns [1, 33].

WMH, already situated at the nexus of multiple structural disadvantages including limited English proficiency, precarious migration or visa status, and the cultural and professional silences surrounding sexual health within the clinical encounter, navigate a healthcare system in which their SF concerns are not only unrecognised but often rendered unintelligible within dominant clinical discourses [19]. For these women, whose trajectories may be marked by displacement, gender-based violence, and prolonged exclusion, the absence of culturally sensitive, trauma-informed, and explicitly articulated conversations about SF does not constitute a minor omission [34]. Providers’ accounts in this study suggest that this absence operates as a form of structural silencing across multiple levels of healthcare, which the adapted ABMHSU framework helps to make visible.

These dynamics are not restricted to the macro-structural level; they materialise most acutely within the clinical encounter itself. Here, both providers and patients operate within a constrained matrix of emotional, cultural, and institutional pressures. The clinical moment where SF could be meaningfully addressed is frequently undermined by provider discomfort, limited training, and perceived cultural inappropriateness, while patients’ disclosures are curtailed by stigma, fear, or prior negative experiences [35]. The result, as providers depicted it, is a patterned silence, mutually sustained yet structurally driven, that inhibits effective therapeutic dialogue about sexual wellbeing.

**Fig. 1:**
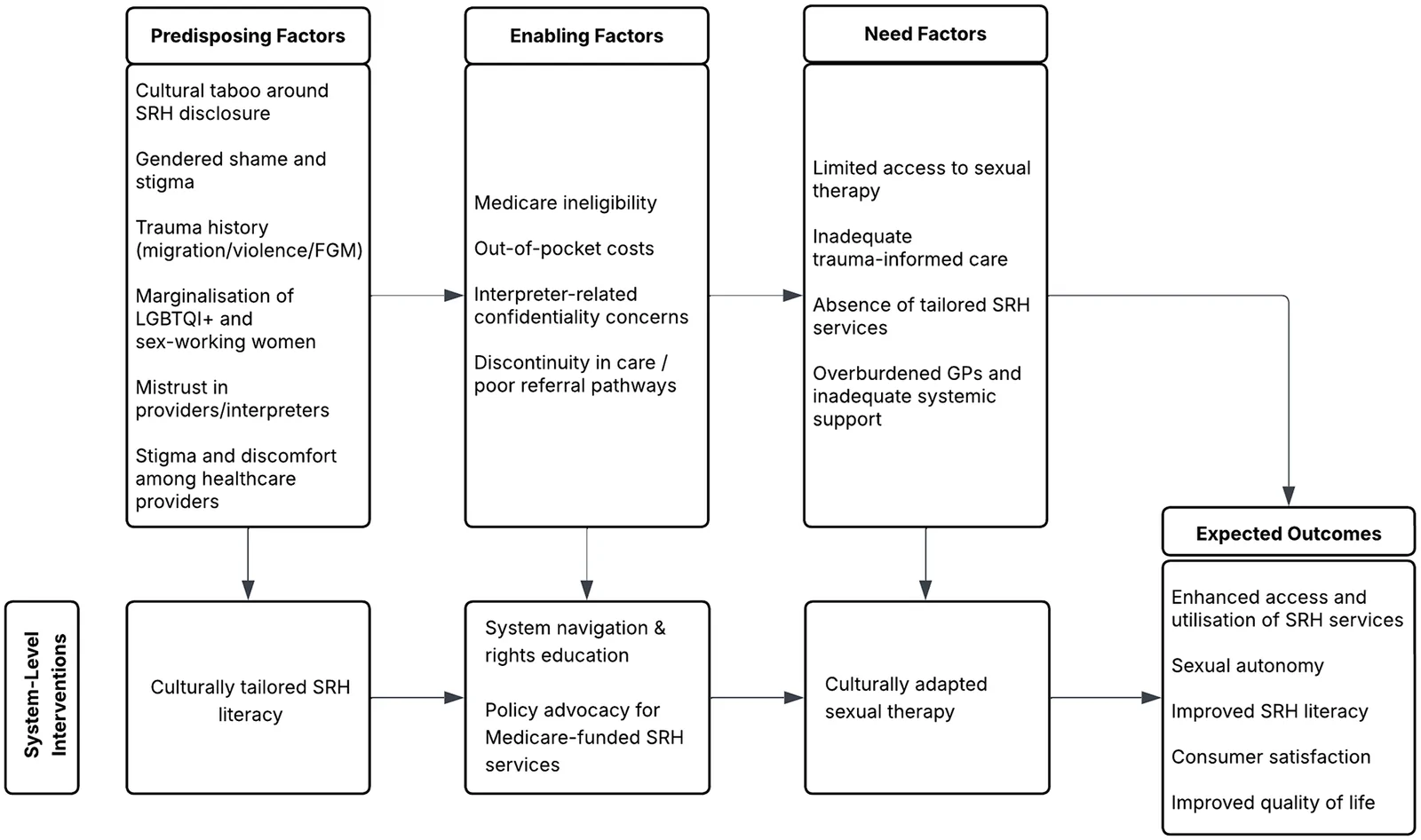
Adapted ABMHSU framework for SRH service use among women with migration history

To synthesise these findings and inform targeted intervention design, this study employed a conceptual framework informed by ABMHSU. Although ABMHSU is conventionally applied to explain individuals’ health service use, in this study it organises providers’ situated observations of the predisposing, enabling, and need-related barriers shaping WMH’s access to and utilisation of SF-related SRH care. The adapted model categorises these barriers into three interrelated domains (Predisposing Factors, Enabling Resources, and Need Factors) while mapping system-level interventions designed to address factors influencing SRH service utilisation among WMH (Fig. 1).

Providers’ accounts in this study point to a bidirectional communication barrier situated within the Predisposing Factors domain of the ABMHSU model. WMH are constrained by cultural taboos, internalised shame, and fear of judgement, while healthcare providers are inhibited by personal discomfort and internalised stigma when addressing sexual concerns [36–38]. This mutual reticence results in a clinical impasse, as both parties defer responsibility for initiating dialogue. Women often expect providers to open such discussions, yet many clinicians hesitate, citing embarrassment and uncertainty about cross-cultural appropriateness [10, 39]. These dynamics are consistent with the REVIVE study, which found that although many women wished to discuss sexual issues, few reported being asked [12]. Addressing these barriers requires culturally tailored SRH literacy interventions designed to normalise sexual wellbeing within diverse cultural frames. Such programmes can enhance provider-patient communication, reduce shame, and support sexual agency, as supported by evidence in multicultural health promotion literature [2, 40–42].

Further challenges are situated within the structural landscape of the health system, aligning with the enabling resources domain. Participants consistently identified the absence of structured, ongoing training in sexual health as a core limitation. Training was described as sporadic, optional, and often reactive, introduced only in response to critical incidents or provider discomfort. Providers reported that this underinvestment in capacity-building constrained their ability to offer culturally safe and trauma-informed care.

These limitations are further compounded by disorganised systems for resource access, including fragmented referral pathways, inconsistent interpretation services, and lack of centralised tools for SRH delivery. Providers reported difficulties navigating an environment lacking in coordination, clarity, and continuity. Similar findings have been reported in other studies, such as the recent systematic review, which identified structural under-resourcing, fragmented interprofessional coordination, and insufficient SRH training as persistent barriers to effective care in diverse populations [43].

To address these enabling constraints, system-level interventions could include structured navigation support, rights-based education, and advocacy for Medicare-funded SRH services. Such measures would not only improve service access for WMH but also reduce the burden on front-line providers. WHO guidance also emphasises the need for culturally responsive, rights-based SRH systems that integrate self-care approaches and strengthen primary care capacity, especially for marginalised and mobile populations [44]. To implement such models effectively, provider training must be comprehensive, mandatory, and supported by accessible, centralised resources that ensure consistency and continuity in SRH care.

Additional barriers identified by providers emerged from the clinical interface, representing the need factors domain of the framework. GPs, typically the first point of contact for MRW, are overextended, facing systemic time pressures and insufficient financial incentives to engage comprehensively with SRH. This strain leads many to deprioritise SF, especially when it falls outside biomedical urgency or funding mechanisms. Participants highlighted that even when providers are motivated to address sexual concerns, their efforts are constrained by a lack of time, specialised training, and institutional support. In this context, the routine integration of SF becomes not only logistically challenging but structurally unsupported. Participants also emphasised that training alone is insufficient. For MRW whose sexual health experiences are shaped by trauma, coercion, or displacement, access to therapeutic care is critical [45]. Yet, sexual therapy remains financially and logistically inaccessible, particularly for women ineligible for Medicare [46]. The introduction of culturally adapted sexual therapy services, publicly subsidised and delivered by trained professionals, is a necessary modifiable intervention to address unmet therapeutic needs. As highlighted by the Multicultural Centre for Women’s Health, effective SRH responses must prioritise cultural safety, linguistic accessibility, and structural equity to ensure that care is meaningful and appropriate for diverse populations [47]. Critically, therapeutic interventions must be culturally grounded. Engagement with MRW cannot rely solely on clinical proficiency; it requires awareness of the sociocultural factors shaping how women interpret, disclose, and seek help for SF-related concerns.

A key strength of this study lies in its purposive inclusion of providers from diverse cultural and professional backgrounds, which contributed to a more nuanced and contextually embedded analysis. Reflexive engagement by the research team further enhanced interpretive rigour and cultural sensitivity.

Nonetheless, several limitations must be acknowledged. First, the study draws on the perspectives of ten healthcare and community service providers, recruited through convenience sampling supplemented by snowball sampling. While iterative team review indicated that the three analytic domains were richly represented in the data, this sampling design produced a sample shaped by accessibility rather than deliberate selection. Self-selection is also likely to have biased the sample toward providers with prior interest in SF, equity, or culturally responsive care; providers who consider SF outside their scope of practice may have been less likely to participate.

Additionally, engagement from senior medical specialists was limited despite extensive outreach. Only one gynaecologist was recruited and no GPs participated. As GPs are typically the first point of contact for SRH care in Australia, this absence constrains the transferability of findings to general practice settings and to the perspectives of medical specialists who routinely manage complex SF presentations.

The study was also conducted within metropolitan SA. Service structures, workforce composition, and the demographics of women with migration histories differ across rural and remote settings and across other Australian jurisdictions, which limits transferability to those contexts.

## Conclusion

This study suggests that SF remains structurally marginalised in the clinical care of WMH, constrained by silences in the clinical encounter, institutional fragmentation, and unmet therapeutic needs as described by providers. Applying the adapted ABMHSU framework, it identifies modifiable barriers across predisposing, enabling, and need-related domains that hinder equitable SRH access.

Key interventions, including culturally tailored SRH literacy, navigation support, and publicly funded, culturally adapted sexual therapy, can facilitate enhanced service utilisation, improved SRH literacy, sexual autonomy, consumer satisfaction, and better quality of life. Achieving these outcomes requires not only provider training and policy reform but a reframing of SF as a central component of health equity.

Future efforts could prioritise the implementation of these strategies and invest in participatory research that centres the voices of WMH in designing inclusive, responsive SRH services.

Confidentiality and anonymity were rigorously maintained throughout the research process. Participant identities were protected by assigning unique identification codes, and all identifying information was removed from transcripts to safeguard privacy. Data were securely stored on password-protected systems of the university accessible exclusively to the approved research team, adhering to institutional data confidentiality and data management protocols.

To acknowledge the time and effort involved, participants received a $100 gift card after the interview. This approach aligns with NHMRC guidelines, which support modest reimbursement for time, effort, and inconvenience, provided it does not constitute coercion or undue influence [29].

## Supplementary Information

Below is the link to the electronic supplementary material.

Supplementary Material 1

## Data Availability

The data generated and analysed during this study are available from the corresponding author upon reasonable request. Due to ethical and legal considerations, access to some data may be restricted to protect participant confidentiality. Data will be shared via a relevant public repository once conditions for anonymisation and de-identification have been met.

## Abbreviations

ABMHSU: Andersen’s behavioural model of health services use
CALD: Culturally and linguistically diverse
EDQ: Exploratory-descriptive qualitative
FGM: Female genital mutilation
FSD: Female Sexual dysfunction
GP: General practitioner
HIV: Human immunodeficiency virus
HPV: Human papillomavirus
HREC: Human research ethics committee
ISSWSH: International society for the study of women’s sexual health
LMICs: Low- and middle-income countries
MCWH: Multicultural centre for women’s health
MRFF: Medical research future fund
NHMRC: National health and medical research council
PBS: Pharmaceutical benefits scheme
QoL: Quality of life
REVIVE: REal women’s views of treatment options for menopausal vaginal changes
RISE: Refugee and immigrant sexual health endeavour
SA: South australia
SF: Sexual function
SRH: Sexual and reproductive health
STI: Sexually transmitted infection
UK: United kingdom
USA: United states of america
VVA: Vulvovaginal atrophy
WHO: World health organisation
WHRTN: Women’s health research, translation and impact network
WMH: Women with migration histories
