# Supplementary material for: Healthcare providers' approaches to sexual function in women with migration histories: a qualitative study using Andersen's behavioural model
Source: Int J Equity Health. 2026 Jun 18;25:211. doi: 10.1186/s12939-026-02917-9 (PMC13584252; doi:10.1186/s12939-026-02917-9)
Supplement: Supplementary file 1 [file 12939_2026_2917_MOESM1_ESM.docx]

# SUPPLEMENTARY FILE 1

**Predisposing Factors**

*(Focus: Cultural, attitudinal, and demographic influences on SRH care engagement)*

**Professional Background**
• What is your professional specialty?
• How long have you been working in your current field?
• How much of your clinical experience involves working with migrant and refugee women? (Can you provide examples of the types of cases you commonly encounter?)

**Common Concerns and Issues**
• Based on your clinical experience, what are the most common concerns or issues related to sexual function among migrant and refugee women?
• Can you provide examples of how these concerns present in clinical settings? (For instance, have patients explicitly raised concerns about sexual function, or do these issues emerge indirectly during broader discussions about reproductive health?)

**Influence of Cultural Values and Beliefs**
• Are there specific cultural factors that you find particularly significant in influencing discussions or care related to sexual function?
• How do cultural values or beliefs, such as stigma, impact the help-seeking behaviours of migrant and refugee women?
• Have you noticed differences based on cultural background or level of acculturation?
• Can you think of any specific instances where cultural norms affected communication or disclosure?

**Challenges in Addressing Sexual and Reproductive Health**
• Do you believe that sharing a similar cultural background or speaking the patient's language influences their level of trust and understanding?
• Can you provide an example of a situation where cultural or linguistic alignment facilitated or hindered communication?

**Enabling Resources**

*(Focus: Structural, policy, or service-related facilitators and barriers)*

**System-Level and Institutional Challenges**
• What challenges have you encountered when addressing sexual or reproductive health concerns with migrant and refugee women? (Consider factors such as language barriers, cultural stigma, patient discomfort, or lack of resources.)
• From a healthcare system perspective, how do these issues (like trust and confidentiality) affect these interactions?
• Can you provide an example of a situation where concerns about confidentiality affected patient communication or engagement?

**Communication Methods and Approaches**
• What strategies or approaches do you use to initiate discussions about sexual function with migrant and refugee women?
• Do you find that certain approaches work better for specific cultural groups?
• Have you received any training on effectively communicating about sexual and reproductive health with culturally diverse patients?
• Can you recall a specific training that was particularly effective or ineffective?
• How do you tailor your communication style to ensure comfort and understanding for patients from diverse cultural backgrounds?
• Can you provide examples of how you adjust your communication based on cultural context?

**Need Factors**

*(Focus: Perceived or evaluated needs and service gaps)*

**Need for Culturally Tailored Training and Resources**
• Are you aware of any tools or resources currently available to support the provision of sexual and reproductive health information to migrant and refugee women?
• Are these resources accessible and practical in your clinical context?
• In your opinion, is there a need for culturally tailored training or resources to improve sexual and reproductive healthcare for this population?
• If yes, what specific areas should these resources focus on?
• What types of training, tools, or resources would be most beneficial for healthcare providers? (e.g., trauma-informed care, sexual function communication, cultural competency)
• How can healthcare services be improved to better meet the cultural and sexual health needs of migrant and refugee women?
• Can you suggest specific programme or policy changes that could address these gaps?
• Are you aware of any existing clinical guidelines or public health policies that address these issues?
• If yes, how effectively are they being implemented in practice?

**Closing Questions**

• Is there anything else you would like to share about your experiences providing sexual and reproductive healthcare to migrant and refugee women?
• Do you have any suggestions for future interviews or questions we may have overlooked?
• Do you know anyone working in this area who might be interested in participating in this research or contributing to policy changes to more effectively address these concerns?
